# Utility of specific amino acid ratios in screening for pyruvate dehydrogenase complex deficiencies and other mitochondrial disorders associated with congenital lactic acidosis and newborn screening prospects

**DOI:** 10.1002/jmd2.12153

**Published:** 2020-08-16

**Authors:** Jirair K. Bedoyan, Rosemary Hage, Ha Kyung Shin, Sharon Linard, Edwin Ferren, Nicole Ducich, Kirkland Wilson, April Lehman, Lori‐Anne Schillaci, Kandamurugu Manickam, Mari Mori, Dennis Bartholomew, Suzanne DeBrosse, Bruce Cohen, Sumit Parikh, Douglas Kerr

**Affiliations:** ^1^ Departments of Genetics and Genome Sciences Case Western Reserve University (CWRU) Cleveland Ohio USA; ^2^ Pediatrics CWRU Cleveland Ohio USA; ^3^ Center for Human Genetics University Hospitals Cleveland Medical Center (UHCMC) Cleveland Ohio USA; ^4^ Center for Inherited Disorders of Energy Metabolism (CIDEM) UHCMC Cleveland Ohio USA; ^5^ Newborn Screening and Radiation Chemistry Ohio Department of Health Laboratory Columbus Ohio USA; ^6^ School of Medicine CWRU Cleveland Ohio USA; ^7^ Nationwide Children's Hospital (NCH) and The Ohio State University College of Medicine Section of Genetic and Genomic Medicine Columbus Ohio USA; ^8^ Department of Pediatrics Akron Children's Hospital (ACH) Rebecca D. Considine Research Institute Akron Ohio USA; ^9^ Northeast Ohio Medical University Rootstown Ohio USA; ^10^ The Cleveland Clinic Foundation (CCF), Neurosciences Institute Cleveland Ohio USA

**Keywords:** alanine, ketogenic amino acids, ketogenic diet, lactic acidosis, mitochondrial disorder, newborn screening, proline, pyruvate dehydrogenase complex deficiency

## Abstract

Pyruvate dehydrogenase complex deficiencies (PDCDs) and other mitochondrial disorders (MtDs) can (a) result in congenital lactic acidosis with elevations of blood alanine (Ala) and proline (Pro), (b) lead to decreased ATP production, and (c) result in high morbidity and mortality. With ~140,000 live births annually in Ohio and ~1 in 9,000 overall prevalence of MtDs, we estimate 2 to 3 newborns will have PDCD and 13 to 14 others likely will have another MtD annually. We compared the sensitivities of plasma amino acids (AA) Alanine (Ala), Alanine:Leucine (Ala:Leu), Alanine:Lysine and the combination of Ala:Leu and Proline:Leucine (Pro:Leu), in subjects with known primary‐specific PDCD due to *PDHA1* and *PDHB* mutations vs controls. Furthermore, in collaboration with the Ohio newborn screening (NBS) laboratory, we determined Ala and Pro concentrations in dried blood spot (DBS) specimens using existing NBS analytic approaches and evaluated Ala:Leu and Pro:Leu ratios from DBS specimens of 123,414 Ohio newborns in a 12‐month period. We used the combined Ala:Leu ≥4.0 and Pro:Leu ≥3.0 ratio criterion from both DBS and plasma specimens as a screening tool in our retrospective review of newborn data. The screening tool applied on DBS and/or plasma (or serum) AA specimens successfully identified three unrelated females with novel *de novo* *PDHA1* mutations, one male with a novel *de novo* X‐linked *HSD17B10* mutation, and a female with *VARS2* mutations. This work lays the first step for piloting an NBS protocol in Ohio for identifying newborns at high risk for primary‐specific PDCD and other MtDs who might benefit from neonatal diagnosis and early institution of known therapy and/or potential novel therapies for such disorders.

**Abbreviations**
CRC: combined Ala:Leu ≥4.0 and Pro:Leu ≥3.0 ratio criterion
DBS: dried blood spot
DCA: dichloroacetate
KD: ketogenic diet
MtD: mitochondrial disorder
NBS: newborn screening
PAA: plasma amino acids
PB: phenylbutyrate
PDCD: pyruvate dehydrogenase complex deficiency
SAA: serum amino acids
TCA: tricarboxylic acid cycle

## 1 INTRODUCTION

Pyruvate dehydrogenase complex (PDC) deficiencies and disorders of pyruvate metabolism are neurometabolic mitochondrial disorders (MtDs) that lead to decreased ATP production and result in high morbidity and mortality. PDCDs are subclassified into at least three groups, primary‐specific, primary‐generalized, and secondary PDCD.^1^ The majority (70%‐90%) of genetically resolved PDCD mutations are due to primary‐specific PDC genes, with those due to *PDHA1* (82%‐88%) predominating.^1^, ^2^, ^3^ More than 500 cases of PDCDs are reported, and many others presumably are not reported or diagnosed, so the actual incidence or prevalence is unknown. However, based on the estimated overall prevalence of MtDs (~1 in 9,000 individuals^4^) and that PDCD is the second most frequent single cause of genetic lactic acidosis^5^ and the second most common MtD within the North American Mitochondrial Disease Consortium (NAMDC) Registry entries (~22%),^6^ we estimate between 1 in 50,000 and 75,000 live births per year in North America will have primary‐specific PDCD. With ~140,000 live births annually in Ohio and overall prevalence of MtDs,^4^ we estimate two to three newborns annually in Ohio will have PDCD and 13 to 14 others likely will have another MtD associated with congenital lactic acidosis, although these could be underestimates if the prevalence of MtDs is significantly higher than previous estimates.^7^

The clinical presentation of PDCD is highly variable and ranges from fatal congenital lactic acidosis and congenital brain abnormalities including corpus callosum abnormalities (15%‐55%), ventriculomegaly (35%‐85%) and Leigh syndrome (12%‐25%), to relatively mild ataxia or neuropathy with normal cognitive function and long survival.^2^, ^8^, ^9^, ^10^, ^11^, ^12^ Epilepsy (16%‐57%), hypotonia (46%‐89%), and developmental delay (57%‐83%) also are other common findings in subjects with PDCD.^2^, ^8^, ^10^, ^11^, ^12^ The best predictor of survival and cognitive outcome in those affected appears to be the age of clinical onset, with neonatal presentations typically associated with early death, and childhood‐onset cases associated with better survival and with normal or mild to severe cognitive disability.^2^ The mean and median ages of diagnosis of childhood‐onset PDCD features are about 31 and 12 months, respectively.^2^, ^13^

Early and correct diagnosis of PDCD and early therapeutic intervention with alternate energy sources (eg, ketogenic diet [KD]^13^, ^14^), activators of residual PDC activity (eg, dichloroacetate^15^ or phenylbutyrate^16^, ^17^, ^18^, ^19^), and/or thiamine supplementation (50‐2000 mg per day^2^, ^20^) could lead to improved developmental and cognitive outcome, quality of life, and survival for infants with primary‐specific PDCD.^21^, ^22^, ^23^ KD use in PDCD has had various positive outcomes and is the therapeutic modality of choice for primary‐specific PDCD.^2^, ^13^, ^14^, ^24^

Patients with impaired mitochondrial PDC function have elevated concentrations of lactate and pyruvate with normal lactate to pyruvate (L:P) ratio or elevated alanine (Ala) concentration, from transamination of accumulating pyruvate, in blood (or CSF).^5^ Besides PDCD, Ala elevation is also observed in other primary MtDs with dysfunctional electron transport chain (ETC) activity, pyruvate carboxylase deficiency, and urea cycle disorders.^5^, ^25^, ^26^ Lactate is a strong marker for defective energy metabolism including those due to PDCD, and L:P ratio reflects cellular NADH/NAD^+^ redox balance and ETC activity,^5^ but lactate is not a metabolite measured in newborn screening (NBS). However, lactate at concentrations commonly observed in both genetic and acquired lactic acidosis inhibits proline oxidase,^27^ the first enzyme of the proline (Pro) degradation pathway, with the rate of Pro degradation varying inversely with increased lactate concentration (ie, the *K*_*m*_ of proline oxidase for Pro increases with increasing concentrations of lactate, with 5 mM lactate inhibiting ~75% of proline oxidase activity in rat liver mitochondria).^27^ Therefore, blood (serum or plasma) Pro would be another marker of lactic acidosis but this likely would not be so for CSF Pro because of known poor permeability of proline across the blood‐brain barrier.^28^, ^29^ Among all the amino acids, only leucine (Leu) and lysine (Lys) are strictly ketogenic amino acids, implying their metabolism would not involve the glycolytic pathway to generate pyruvate but instead would enter the tricarboxylic acid cycle at the acetyl‐CoA level bypassing PDC. Because Leu and Lys concentrations are not influenced by the amount of lactate, Ala or Pro produced from impaired PDC function, they can in principle be used as normalizing metabolites in quantitative analysis of Ala and Pro.

We sought to investigate the utility of specific amino acid ratios (particularly Ala:Leu and Pro:Leu) in screening PDCDs and other MtDs in order to identify patients who may benefit from early diagnosis and therapeutic intervention(s). This work lays the foundation for piloting an NBS protocol in Ohio for identifying newborns at high risk for such disorders, particularly primary‐specific PDCD.

## 2 MATERIALS AND METHODS

In addition to PAA (or SAA) and NBS dried blood spot (DBS) data, blood (or CSF) lactate and pyruvate concentrations, L:P ratio, and PDC assay data (if performed) as well as brain imaging and clinical course information were also reviewed. Molecular genetic testings were sent to CLIA‐ and/or CAP‐approved clinical laboratories. Gestational age (GA) categories for preterm, term and postterm newborns were as follows: Extreme and very preterm, ≥22 and ≤33 weeks; late preterm, ≥34 and ≤36 weeks; early‐, full‐, and late‐term, ≥37 and ≤41 weeks; and postterm ≥42 weeks.^30^, ^31^

### 2.1 Ohio NBS laboratory data

Deidentified Ala, Pro, and Leu concentration data from 136,282 DBS specimens on filter paper collected from newborns screened over a 1‐year period between November 15, 2018 and November 14, 2019, were provided by the Ohio NBS laboratory in Columbus, OH. Lysine is *not* measured at the Ohio NBS Laboratory. Deidentified data also included newborn information such as gender, GA (weeks), birth weight (BW, g), NICU stay (yes/no), total parenteral nutrition (TPN) usage (yes/no), and age at time of collection of DBS specimen (hours of life [HOL]). Ala, Pro, and Leu concentration data from specimens with incomplete or missing newborn characteristic data were excluded. “Repeat NBS” test specimens (1,618 or 1.2% of original 136,282) were excluded. We also limited NBS data to newborns with GA of ≥22 and ≤43 weeks and those with DBS specimen collected at ≥24 and ≤48 HOL. Newborns who were noted to be on TPN when the DBS specimen was collected (1,651 specimens; 1.2%) or TPN usage information was missing (2,729 specimens; 2.0%) also were excluded. In total, data from 12,868 specimens were excluded (9.4% of original 136,282). Therefore, data from the remaining subset of 123,414 nonduplicate and data‐complete (with the exception of 4,426 [3.6%] specimens which had no NICU‐stay information recorded) specimens (ie, 90.6% of the initial 136,282) were used in subsequent analysis.

Measurements of Ala, Pro, and Leu concentrations in DBS specimens were made in multiple reaction monitoring mode using Waters triple quadrupole tandem mass spectrometers. Data were processed by MassLynx software (Waters; Milford, Massachusetts) loaded onto the instruments and then transferred through an algorithm specified in the PerkinElmer Specimen Gate laboratory information management system for data review and reporting. The Neobase Non‐derivatized MSMS Kit (3040‐001U, 3041‐0020; PerkinElmer, Turku, Finland) was used according to manufacturer instructions. Flow injection was used to analyze 20 μL samples of eluate from 3 mm DBS punches to which known concentrations of internal standards were added. Amino acid concentrations in these samples were estimated from the ratio of measurements of amino acid to internal standard. Leu peaks are not resolved from those for isoleucine and hydroxyproline in this test system.

### 2.2 Statistical analysis

Means of subject Ala (μM), Ala:Leu, and Ala:Lys values were compared to controls using Welch's two‐sample *t*‐test for unpooled variances with one‐tailed significance level of *P* < 0.05. The R Project for Statistical Computing (https://www.r-project.org),^1^ a free software environment for statistical computing and graphics, was used for many statistical analyses. Linear regression was used to approximate the relationship between Pro:Leu and Ala:Leu ratios, with Ala:Leu and Pro:Leu as the independent and dependent variables, respectively. Results of regression analysis were tested for significance using the *F*‐statistic test.^32^ Smoothed confidence intervals (CI) for the regression analysis were displayed with scatter plots using ggplot2 library (https://ggplot2.tidyverse.org) in R. Additionally, Welch's two‐sample *t*‐test for unpooled variances was again used with one‐tailed significance level of *P* < 0.05 to compare mean ages of subjects falling inside and outside the CI on the plots. Also, the two‐tailed two‐sample with means and unequal variances *z*‐test and the chi‐squared test of independence in Microsoft Excel with a significance level of *P* < 0.05 were also used to determine differences and relationships, respectively, between two groups of data.

## 3 RESULTS

### 3.1 Combination amino acid ratios in subjects with PDCD

Plasma Ala, Pro, Leu, and Lys from 20 subjects enrolled in our IRB‐approved study with *PDHA1* mutations (12 females and 8 males) and 2 compound heterozygote subjects with *PDHB* mutations (1 female and 1 male) were analyzed (Supplementary Table S1). Average of two PAA samples per subject (min 1 and max 5, n = 43) were collected before initiation of KD. Mean and median ages of the PDC deficient subjects were 5 years and 1 year, respectively (min and max: 3 days and 32 years, respectively), while controls with normal PAA were 14 and 7 years, respectively (min and max: 44 days and 50 years, respectively, n = 10).

**FIGURE 1:**
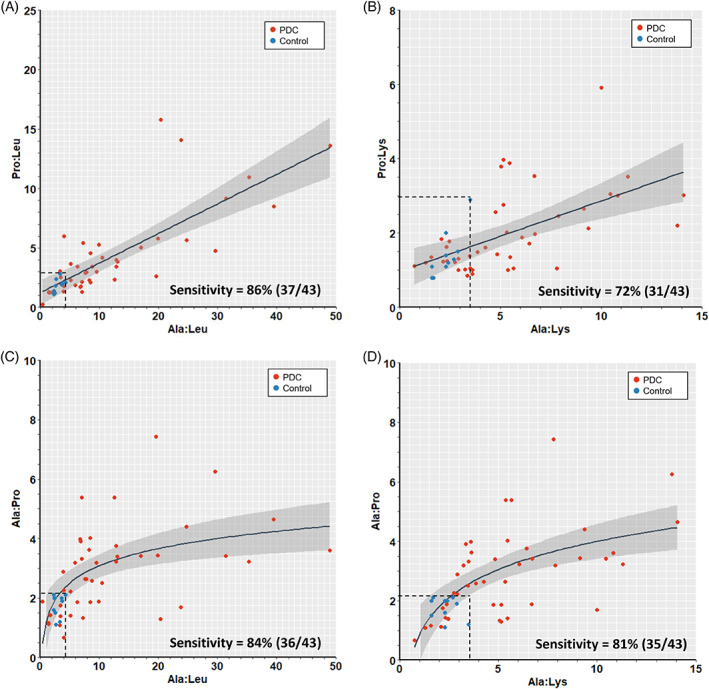
Plots and regression analyses of plasma amino acid (PAA) ratios for known pyruvate dehydrogenase complex deficiency (PDCD) cases and normal controls. A, Pro:Leu vs Ala:Leu and, B, Pro:Lys vs Ala:Lys plots and linear regressions are shown. C, Ala:Pro vs Ala:Leu and D, Ala:Pro vs Ala:Lys plots and logarithmic regressions are shown. Sensitivities for identifying subjects with PDCD for the pairs of ratios using the cut‐offs (dotted lines) are shown. In the key on the graphs, PDCD subjects (red dots) and normal controls subjects (blue dots). The gray shaded areas correspond to 95% confidence interval (CI) for the regression

Hyperprolinemia is generally noted with lactic acidosis. Not surprisingly, a linear relationship (regression) between Pro:Leu and Ala:Leu is noted with PAA (Figure 1A) with high F‐statistic (59) and statistical significance (*P* < 0.0001). In contrast to the relatively low sensitivities noted for Ala, Ala:Leu and Ala:Lys (67%‐77%; Supplementary Results S1 [Appendix S1], Supplementary Figure S1 and Supplementary Table S1), the Pro:Leu and Ala:Leu pair with cut‐offs Pro:Leu ≥2.8 and Ala:Leu ≥4.3 together provided a higher sensitivity (86%) for identifying subjects with primary‐specific PDCD, even when compared to the Pro:Lys and Ala:Lys pair with PAA (Figure 1B). A logarithmic relationship between Ala:Pro and Ala:Leu (Figure 1C) as well as Ala:Pro and Ala:Lys (Figure 1D) was noted with F‐statistic (18 and 22, respectively) and statistical significance (*P* = 0.0001 and *P* < 0.0001, respectively). A sensitivity of 89% was observed for the Pro:Leu and Ala:Leu combination when PDC deficient subjects with *PDHA1* mutations only were analyzed (data not shown). Therefore, based on the above determination of sensitivities for the paired ratios in genetically‐resolved PDC deficient subjects, we chose *preliminary* cut‐offs (rounded to significant digit) of Ala:Leu ≥4.0 and/or Pro:Leu ≥3.0 in our subsequent analyses.

We also determined that subjects with data landing outside the 95% CI were 5 to 6 years older at time of PAA analysis than those within the 95% CI and this was statistically significant across other paired amino acid ratio plots, such as Ala:Pro vs Ala:Leu, Pro:Lys vs Ala:Lys, and Ala:Pro vs Ala:Lys (data not shown; also Supplementary Results S2 [Appendix S1]).

### 3.2 Application of the combined Ala:Leu ≥4.0 and Pro:Leu ≥3.0 ratio criterion (CRC) on a cohort of patients, comparing DBS amino acids with PAA

**FIGURE 2:**
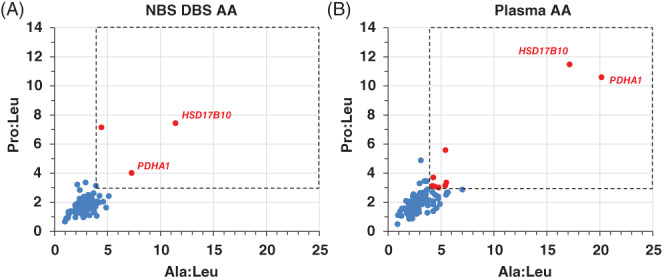
Pro:Leu vs Ala:Leu plots for a cohort of 104 patients born in Ohio and followed at one institution (UHCMC). A, Newborn screening (NBS) dried blood spot (DBS) and, B, plasma specimens used for plasma amino acids (PAA) analysis, respectively. Subjects shown in red dots satisfy the combined Ala:Leu ≥4.0 and Pro:Leu ≥3.0 criterion (combined Ala:Leu ≥4.0 and Pro:Leu ≥3.0 ratio criterion [CRC]; dashed rectangle). The responsible mutated gene in subjects (red dots) with confirmed pathogenic variants is noted. Among the 104 patients (55 males, 49 females), the average ± SD (min, max) of gestational age, birth weight, DBS collection time (hours of life [HOL]), and time of first plasma draw for amino acids analysis (days of life [DOL]) were 35 ± 5 weeks (23, 41), 2,527 ± 969 g (525, 4,225), 28 ± 6 HOL (2, 51), and 72 ± 102 DOL (1, 503), respectively

The Ohio NBS laboratory by agreement has been releasing deidentified Ala and Pro concentrations from NBS DBS specimens of newborns born in Ohio since May 4, 2018. We reviewed specific neonatal and biochemical testing characteristics of 104 patients followed at our institution (UHCMC) and born between May 5, 2018 and November 14, 2019 in Ohio, in order to determine how many met combined Ala:Leu ≥4.0 and Pro:Leu ≥3.0 ratio criterion (CRC) by DBS or plasma testing (Figure 2 and Supplementary Table S2). The patients had *not* received TPN, which has a relatively high concentration of leucine, nor initiated KD at time of DBS specimen collection or at *first* plasma sample collection for amino acids analysis. Three patients by DBS (Figure 2A red dots) and nine patients by PAA (Figure 2B red dots) met CRC from analysis of DBS and plasma samples, respectively (Supplementary Table S2). However, only two patients fulfilled CRC for both sample types; one female newborn diagnosed with PDCD due to a novel heterozygous pathogenic *PDHA1* mutation (Met294Ilefs*4) and was started KD at around 5 weeks of age, and one male newborn diagnosed with MtD due to a novel X‐linked hemizygous pathogenic *HSD17B10* mutation (Arg29Gly), where the gene encodes an essential component of mitochondrial RNase P required for processing of mitochondrial DNA transcripts. The latter patient died at 6 months of age following status epilepticus. Therefore, of the 104 patients referred to UHCMC, only three fulfilled CRC by DBS testing and flagged as high risk for PDCD or another MtD by NBS. Follow‐up PAA screening resulted in two of them (2/3) satisfying CRC again, and consequently requiring further confirmatory functional (enzymatic) and/or molecular testing for PDCD and/or other MtDs associated with congenital lactic acidosis. Furthermore, 3/5 patients in the NICU at time of DBS collection and fulfilling CRC were term newborns (Supplementary Table S2).

### 3.3 Characteristics of newborns with specific amino acids and amino acid ratios from NBS data

**FIGURE 3:**
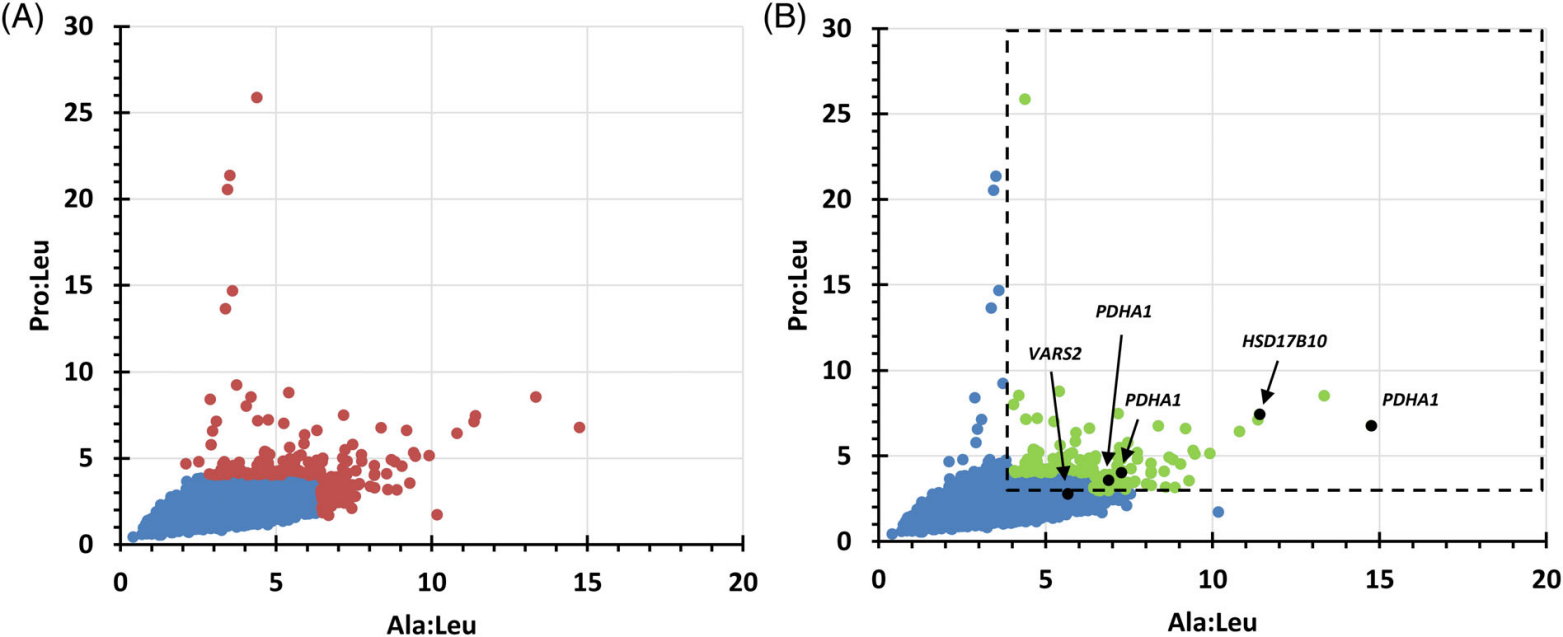
Pro:Leu vs Ala:Leu plots of newborn screening (NBS) dried blood spot (DBS) data for the population of deidentified screened newborns born in Ohio over a 1‐year period, with a subset of newborns with pyruvate dehydrogenase complex deficiency (PDCD) or another MtD noted. Distribution of DBS data from nonduplicate and data‐complete newborn specimens <99.9%ile for Ala:Leu or Pro:Leu (3A, 123,197 blue dots), ≥99.9%ile for Ala:Leu or Pro:Leu (3A, 217 red dots), and ≥ 99.9%ile for *either* Ala:Leu or Pro:Leu ratios and *also* fulfilling combined Ala:Leu ≥4.0 and Pro:Leu ≥3.0 ratio criterion (CRC) (3B, 145 green dots within the dashed rectangle from among the total 123,414  DBS data points). Subjects molecularly confirmed with PDCD or another MtD shown also as black dots

Data from 123,414 nonduplicate and data‐complete newborn specimens were evaluated (Figure 3). Two hundred and seventeen (217) newborns with ≥99.9%ile for *either* Ala:Leu or Pro:Leu (constituting ~0.18% of all specimens; Figure 3A red dots), with 49% of newborns were in the NICU at time of DBS collection and 78% were term newborns (early, full and late). Because the combined Ala:Leu ≥4.0 and Pro:Leu ≥3.0 showed high sensitivity for identifying primary‐specific PDC deficient subjects (see Section 3.1), we determined the number and distribution of subjects who were ≥ 99.9%ile for *either* Ala:Leu or Pro:Leu and who *also* fulfilled CRC by exceeding *both* AA ratios (145 subjects constituting ~0.12% of all specimens; Figure 3B green dots), with 51% of newborns were in the NICU at time of DBS collection and 75% were term newborns (early, full, and late). We also determined that the mean values for GA, BW, Ala, Pro, Ala:Leu, and Pro:Leu for newborns with ≥99.9%ile for either Ala:Leu or Pro:Leu (Figure 3A red dots) were not significantly different than for those with ≥99.9%ile for *either* Ala:Leu or Pro:Leu and who *also* fulfilled CRC (Figure 3B green dots) (Supplementary Results S3 [Appendix S1]). This suggests that it is valid to use DBS specimen data from either newborn groups (red dots or green dots) in subsequent analyses.

Of the 123,414 newborns, 9,275 were in the NICU at time of DBS specimen collection (7.5% of all subjects), while 109,713 were not (88.9% of all subjects) with NICU‐stay not recorded in 4426 (3.6%) newborns. Among 134 419 specimens with gender information, the male to female ratio was 1.04, which was also the same among the 123,414 nonduplicate and data‐complete newborn DBS specimens. Chi‐squared testing of independence showed significantly (*P* = 0.002) more newborn males in the NICU at time of DBS specimen collection and who also were ≥99.9%ile (or ≥99.0%ile) for either Ala:Leu or Pro:Leu with male to female ratio of 2.2 (or 1.6), respectively (data not shown). The reason for this male predominance with abnormally elevated Ala:Leu and/or Pro:Leu is unclear, but may reflect observed gender differences in mitochondrial (dys)function and energy metabolism.^33^

Not surprisingly, newborns not in the NICU at time of DBS collection were predominantly (94.8%) term newborns, while late, very and extreme preterm newborns together constituted ~5.0% of newborns (Supplementary Results S3 [Appendix S1]). The percentage of term newborns in the NICU with ≥99.0%ile for either Ala:Leu or Pro:Leu (64%) was significantly greater than the percentage of term newborns in the NICU at time of DBS collection (46%, *P* < 0.0001; Supplementary Results S3 [Appendix S1]). These results suggest that the etiology of lactic acidosis (as reflected by abnormal elevation of Ala, Pro, Ala:Leu, and/or Pro:Leu in a DBS specimen or plasma) in 60% to 65% of newborns in the NICU may not be due to prematurity, but rather due to other reasons such as sepsis, a genetic/metabolic disorder, cardiopulmonary dysfunction, hypoxemia, or some other condition leading to tissue hypoxia.

### 3.4 Identification of newborns with PDCD or other MtDs using CRC

By engaging a number of geneticists and neurologists across Ohio including those at three NAMDC sites in Ohio (UHCMC/CWRU, CCF, and ACH) and NCH, we identified Ohio newborns diagnosed with PDCD and other MtDs born in the 1‐year period between November 2018 and November 2019, and, retrospectively, evaluated their Ala:Leu and Pro:Leu among other metrics (Figure 3, Table 1 and Supplementary Table S3). We identified five subjects (4 females, one male), three with PDCD and two with MtD, all born at term and in the NICU at time of DBS collection, with BW ranging 2,155 to 3,080 g (Table 1). All five subjects satisfied CRC for plasma or serum specimens, while 4/5 satisfied CRC for DBS specimens (Table 1). All three newborns identified with PDCD satisfied CRC for both DBS and PAA testing (Table 1). Subject 4 was only just below the Pro:Leu ≥3.0 cut‐off (Table 1) and was considered a false negative (FN) by DBS only. Biochemical work‐up for two PDC deficient subjects (Table 1, subjects 1 and 3) were initiated at the time of NICU admission immediately after birth; they were diagnosed with PDCD and started KD before 5 weeks of age. Subject 5 (Table 1) was prenatally noted to have microcephaly and brain abnormalities and was subsequently diagnosed with sensorineural hearing loss after a failed newborn hearing screen. She was initially admitted to a NICU for monitoring PO intake because of known brain anomalies. Her biochemical and genetic work‐up was initiated during a later PICU admission for seizure, hypothermia, and encephalopathy at 3 months of age. She required intubation for apnea and died after redirection of care and thus KD was not initiated. Four subjects (Table 1, subjects 1‐3) were among the 145 subjects with ≥99.9%ile for either Ala:Leu or Pro:Leu and who also fulfilled CRC (Figure 3B). Of the 145 subjects, 109 (75%) were term (includes early, full and late) newborns. These results imply that *at least* 3.7% (4/109) of term infants fulfilling screening parameters (ie, ≥99.9%ile for either Ala:Leu or Pro:Leu, and CRC) have either PDCD or another MtD associated with congenital lactic acidosis.

**TABLE 1:** Subjects with PDCD or MtD identified using the protocol^a^

Subject	Sex	GA (wk)	BW (g)	NBS DBS				Blood AA				Dx	Gene with pathogenic variant(s)	Abn brain MRI or US	Blood		kd	Living (age at death)
				Ala	Pro	Ala:Leu	Pro:Leu	Ala	Pro	Ala:Leu	Pro:Leu				Max lactate (mM)	L:P ratio		
1	F	38	2155	906	415	**14.8**	**6.8**	859	346	**26.0**	**10.5**	PDCD	*PDHA1 c.1024C* > T p.Arg342*	Y	12.6	ND	Y	Y
2	M	38	3060	1348	879	**11.4**	**7.4**	1354	907	**17.1**	**11.5**	MtD	*HSD17B10 c.85C* > *G p.Arg29Gly*	ND	10.8	21	NA	N (6 mo)
3	F	39	3080	370	205	**7.3**	**4.0**	947	498	**20.1**	**10.6**	PDCD	PDHA1 c.874_881dup p.Met294Ilefs*4	Y	7.8	9	Y	Y
4	F	39	2897	357	175	5.6	2.8	513	252	**9.5**	**4.7**	MtD	*VARS2* c.1925del p.Leu642Argfs*48 /c.721C > T p.Arg241Trp	N	15.3	ND	Y	N (7 wk)
5	F	39	2490	569	295	**6.9**	**3.6**	409	318	**13.2**	**10.3**	PDCD	*PDHA1 c.899* + *2T* > *A splice‐site*	Y	15.7	ND	NA^b^	N (3 mo)

*Note*: Numbers in bold and underlined indicate fulfillment of the CRC. Amino acids concentrations in μM.

Abbreviations: Abn, abnormal; BW, birth weight; CRC, combined Ala:Leu ≥4.0 and Pro:Leu ≥3.0 criterion; DBS, dried blood spot; DX, diagnosis; F, female; g, gram; GA, gestational age; KD, ketogenic diet; mo, month; L:P ratio, lactate to pyruvate ratio; M, male; MtD, mitochondrial disorder; NA, not applicable; NBS, newborn screening; N, no; ND, not done; blood AA, plasma or serum amino acids; PDCD, pyruvate dehydrogenase complex deficiency; US, ultrasound; wk, week; Y, yes.

^a^ All subjects were in the NICU at time of DBS collection.

^b^ Subject died after redirection of care and thus KD was not initiated.

For subjects in Table 1, we also evaluated other amino acids and amino acid ratios (Supplementary Table S4) reported to discriminate primary MtDs from PDCD,^25^ including citrulline which can be low in several primary MtDs such as MELAS^34^ and mitochondrial ATP synthase deficiency.^35^ Mean concentrations of citrulline in DBS and blood AA were higher in subjects with MtD (2 and 4) compared to those with PDCD (1, 3, and 5) consistent with others.^25^

## 4 DISCUSSION

Genetic disorders associated with congenital lactic acidosis include PDCD and other MtDs.^36^, ^37^ Such disorders are diagnostic challenges and are usually associated with high morbidity and mortality. Delayed and difficult diagnosis of congenital PDCD is currently prevalent.^2^, ^13^ PDCD is sub‐classified into at least three groups and 35 genes are currently known to be associated with PDCD^21^ (see Supplementary Table S5 for an updated list). Primary‐specific PDCD due to *PDHA1* (>80%) is by far the most common type of PDCD^1^, ^2^, ^3^ for unclear reasons. The incidence of PDCD is estimated to be 1 in 50,000 to 75,000 births annually and we succeeded in identifying three patients with PDCD in a 1‐year period in Ohio.

Statewide collaborations with geneticists and neurologist have identified five patients with PDCD (3) and other MtDs (2) who were born in Ohio within the 1‐year period of this study (Figure 3B; Table 1 and Supplementary Table S3). This frequency is about 63% less than the expected 13 to 14 total newborns with MtDs annually in Ohio, although the frequency of newborns with PDCD was as expected. The number of MtDs is likely fewer than expected because the biochemical and/or molecular diagnoses of the remaining 141 subjects (74 in NICU and 67 *not* in NICU at time of DBS collection) fulfilling screening parameters remain to be determined. All five subjects identified were in the NICU at time of DBS specimen collection. This screening approach may be favoring newborns with PDCD or other MtDs who are markedly symptomatic at birth or have concerning perinatal clinical findings requiring a NICU admission, but this remains to be determined as the identities of more subjects fulfilling CRC are elucidated. It is possible that PDC deficient subjects with milder phenotype and/or no brain anomalies may turn out as FNs by DBS, but this remains to be determined. The X‐linked form of PDCD (due to *PDHA1*) may be variably expressed in cells and tissues in females, which could result in FN ratios by DBS. Lack of perinatal metabolic decompensation and/or lactic acidosis may result in FN ratios by DBS, but true positive (TP) when blood (for PAA or SAA) is collected later during metabolic decompensation and/or lactic acidosis. Genotype may be a reason why some newborns may present as FN by DBS but TP at a later date (ie, late‐onset subjects). Finally, problems with DBS cards or processing of a specimen may sometimes account for a FN result. This work also suggests that the etiology of lactic acidosis in the majority of newborns in the NICU may not be due to prematurity but due to other causes including sepsis, hypoxemia, PDCD, or other MtDs.

The calculated positive predictive value (PPV)^2^ for this screening approach was 2.8% (4/145), with 80% (4/5) sensitivity (with one FN). However, this PPV value could be an underestimate with a more accurate value remaining to be determined as the identities of the majority of outstanding 141 subjects (Figure 3B, green dots) are resolved. By comparison, PPVs in Ohio for Krabbe disease, Hurler syndrome, and Pompe disease, which are on the NBS testing panel, were 1.0% (3/290), 3.3% (3/91), and 18.5% (5/27), respectively, between July 1, 2016 and October 31, 2019.

Ala:Lys was found to be significantly increased in subjects with PDCD (n = 3; 1 on KD and 2 not on KD) vs controls (n = 14), but not so with certain primary MtDs vs controls.^25^ Consistent with Clarke et al, we also found Ala:Lys to be significantly increased in subjects with PDCD (n = 20; all subjects not on KD at time of analysis) vs controls (n = 10) (Supplementary Figure S1). To our knowledge, Ala:Leu, Pro:Leu, and Pro:Lys ratios have not been evaluated before in PDCD. We determined that plasma Ala, Ala:Leu ratio and Ala:Lys ratio are significantly higher in subjects with PDCD vs controls but that the combination of Ala:Leu and Pro:Leu ratios had the highest sensitivity for subjects with PDCD (Figure 1 and Supplementary Figure S1). Leu and Lys are strictly ketogenic amino acids bypassing PDC for their metabolism and thus their utility in normalization with Ala or Pro concentrations to identify subjects with primary‐specific PDCD and other MtDs that disrupt NADH:NAD^+^ redox balance impacting L:P and consequently Pro:Ala (Table 1). However, a notable difference between Leu and Lys is that during catabolism plasma Lys concentration tends to decrease, which is in contrast to Leu and other branched‐chain amino acids which tend to increase in plasma during catabolic states. This may limit their utility when used as denominators in ratios with Ala or Pro with certain metabolic states. Furthermore, certain mitochondrial conditions like dihydrolipoamide dehydrogenase (DLD or E3) deficiency (also classified as primary‐generalized PDCD; Supplementary Table S5), which results in Leu elevation may be missed if Ala:Leu, Pro:Leu, or the combination ratios are used as screening tools.

In Ohio and many other NBS laboratories around the United States, test systems are not configured to capture measurements of several additional biomarkers, such as Lys, not currently used as primary markers in NBS. Where test systems are FDA‐approved, reconfiguring them presents the challenges of off‐label use if additional markers are added and software reconfigurations may not be possible. Furthermore, at this time, the precise impact of the Leu peak not being resolved from isoleucine and hydroxyproline by the testing system used in the Ohio NBS Laboratory on DBS specimens is unclear. It is possible that this has an effect on test sensitivity but this remains to be determined and may be better determined after resolving the diagnosis of more subjects meeting CRC. The mean hydroxyproline and isoleucine concentrations in subjects with PDCD are higher and lower than controls, respectively.^25^ Therefore, it is possible that the effects of isoleucine and hydroxyproline within the leucine peak from DBS specimens cancel out in subjects with PDCD.

Early screening for conditions with acceptable treatments has been a fundamental Wilson and Jungner criterion in guiding the selection of condition(s) for NBS.^38^ Whether it is appropriate to screen for a condition where acceptable treatment(s) may not be applicable as is the case for most primary MtDs (eg, conditions of subjects 2 and 4 in Table 1) may be debatable and might be revisited in the current genomics age.^39^, ^40^ While ethical, social, and legal implications limit use of advanced diagnostic DNA sequencing technologies (eg, whole exome or genome sequencing) at a population level in the context of NBS,^41^ existing NBS analytical approaches could potentially still be used to screen for conditions with known therapeutic options not currently on the expanded NBS panel such as PDCD, and potentially incorporating second‐tier targeted molecular testing in order to increase specificity for primary‐specific PDCD.

## 5 CONCLUSIONS

To our knowledge, this is the first regional (statewide) effort in successfully identifying newborns with PDCD (or other MtDs) using the combination of Ala:Leu and Pro:Leu ratios. Ala and Pro concentrations in DBS specimens are not measured in most NBS laboratories, but this is easily accomplished using existing NBS analytical approaches at relatively little or no cost. The Ohio NBS lab has been providing Ala and Pro concentration data since May 2018 because of this work. More concerted effort by multiple state NBS labs in measuring Ala, Pro, and Leu concentrations in DBS specimens could help validate the utility of using the combination ratios (at current or different cut‐off ratios) and identify newborns with PDCD and other MtDs for early intervention with available and novel therapeutic options. In summary, further investigation of this screening approach, its feasibility, and outcomes would be beneficial and informative to NBS programs.

## CONFLICT OF INTEREST

The authors declare no potential conflict of interest.

## AUTHOR CONTRIBUTIONS

Jirair K. Bedoyan, Rosemary Hage, Ha Kyung Shin, Sharon Linard, Edwin Ferren, Nicole Ducich, and Kirkland Wilson were involved in data collection. Jirair K. Bedoyan, Ha Kyung Shin, Nicole Ducich, and Kirkland Wilson were involved in data analysis. Jirair K. Bedoyan, April Lehman, Lori‐Anne Schillaci, Kandamurugu Manickam, Mari Mori, Dennis Bartholomew, Sumit Parikh, Suzanne DeBrosse, and Douglas Kerr were involved in subject recruitment and enrollment for this work. Jirair K. Bedoyan, Sumit Parikh, and Bruce Cohen are collaborators and NAMDC site PIs in Ohio. Jirair K. Bedoyan wrote the first draft, Jirair K. Bedoyan and Douglas Kerr reviewed and revised the manuscript, and Jirair K. Bedoyan approved the final version as submitted. All authors approved the final manuscript as submitted and agree to be accountable for all aspects of the work. All authors confirm the absence of previous similar or simultaneous publications.

## ETHICS APPROVAL AND INFORMED CONSENT

Informed consent was obtained from subjects or parents/guardians of subjects for inclusion in the University Hospitals Cleveland Medical Center (UHCMC) IRB‐approved “Disorders of Pyruvate Metabolism” study (03‐06‐39) and UHCMC IRB‐approved protocols “Pilot Pyruvate Dehydrogenase Complex Deficiencies (PDCDs) Newborn Screening (NBS) Study” (STUDY20190841) and UHCMC IRB‐approved “Comparison of Methodologies for Amino Acids Analysis from Blood Products” (STUDY20191215). All procedures followed were in accordance with the ethical standards of the responsible committee on human experimentation (institutional and national) and with the Helsinki Declaration of 1975, as revised in 2000.

## ANIMAL RIGHTS

This article does not contain any studies with animal subjects performed by any of the authors.

## Supporting information


**Appendix S1.** Supporting Information (Supplementary Results S1, S2 and S3)

**Supplementary Figure S1** Distribution of plasma Ala, Ala:Leu, and Ala:Lys from PDC deficient subjects due to *PDHA1* (20) or *PDHB* (2) mutations and normal controls. A) Ala (μM), B) Ala:Leu, and C) Ala:Lys from PDC deficient subjects (red dots) and controls (blue dots). Mean and 2 SD (SD) bars shown. Number of false negative (FN) between the maximum value for controls (blue dotted line) and minimum value for PDC deficient subjects (red dotted line) shown. Percentage difference of mean (%Δ), for Ala, Ala:Leu and Ala: Lys metrics were 75%, 122% and 81% respectively. P values were obtained from two‐sample *t*‐test for un‐pooled variances with one‐tailed significance level of *P* < 0.05. ***, *P* < 0.0005.

**Supplementary Table S1** Characteristics of subjects with primary‐specific PDCD not on KD

**Supplementary Table S2** Neonatal and biochemical characteristics of patients fulfilling combined Ala:Leu ≥4.0 and Pro:Leu ≥3.0 criterion (red) from either DBS or plasma analysis at one institution

**Supplementary Table S3** Clinical and molecular details of subjects with PDCD and MtDs identified using the protocol

**Supplementary Table S4** Various amino acids and amino acid ratios of subjects with PDCD and MtD identified using the protocol

**Supplementary Table S5** Sub‐classification of PDCDs: Currently known, possible and potential molecular etiologies of impaired pyruvate oxidation

## Endnotes

^1^ R Core Team. R: A language and environment for statistical computing. R Foundation for Statistical Computing, Vienna, Austria. 2018.

^2^ Positive predictive value (PPV $=\frac{\text{true positive}}{\text{true positive}+\text{false positive}}$), focuses on subjects with a positive screening test in order to evaluate the probability of disease for those subjects.
