# Supplementary material for: Utility of specific amino acid ratios in screening for pyruvate dehydrogenase complex deficiencies and other mitochondrial disorders associated with congenital lactic acidosis and newborn screening prospects
Source: JIMD Rep. 2020 Aug 16;56(1):70–81. doi: 10.1002/jmd2.12153 (PMC7653239; doi:10.1002/jmd2.12153)
Supplement: Supplementary file 1 — Appendix S1. Supporting Information (Supplementary Results S1, S2 and S3) [file JMD2-56-70-s001.docx]

**Supplementary Table 1. Characteristics of subjects with primary-specific PDCD not on KD**

|  |  |  |  |  |  |  |  |  |  |  |  |  |  |  |  |  |  |
| --- | --- | --- | --- | --- | --- | --- | --- | --- | --- | --- | --- | --- | --- | --- | --- | --- | --- |
| **Pt #** | **Gender** | **Zygosity** | **Gene** | **Exon** | **Base Change** | **AA Change** | **Sample type** | **Age at AA analysis (days)** | **ALA** | **LEU** | **LYS** | **PRO** | **ALA:LEU** | **PRO:LEU** | **ALA:LYS** | **PRO:LYS** | **ALA:PRO** |
| 1 | F | Het | PDHA1 | 1 | c.58-1G>A | splice acceptor mut | Plasma | 346 | 822 | 107 | 193 | 312 | 7.7 | 2.9 | 4.3 | 1.6 | 2.6 |
| 1 | F | Het | PDHA1 | 1 | c.58-1G>A | splice acceptor mut | Plasma | 447 | 446 | 87 | 153 | 201 | 5.1 | 2.3 | 2.9 | 1.3 | 2.2 |
| 2 | M | Hemi | PDHA1 | 3 | c.214C>T | p.R72C | Plasma | 544 | 812 | 85 | 103 | 254 | 9.6 | 3.0 | 7.9 | 2.5 | 3.2 |
| 2 | M | Hemi | PDHA1 | 3 | c.214C>T | p.R72C | Plasma | 5275 | 485 | 62 | 91 | 184 | 7.8 | 3.0 | 5.3 | 2.0 | 2.6 |
| 3 | F | Het | PDHA1 | 3 | c.225G>T | p.E75D | Plasma | 341 | 686 | 95 | 136 | 516 | 7.2 | 5.4 | 5.0 | 3.8 | 1.3 |
| 3 | F | Het | PDHA1 | 3 | c.225G>T | p.E75D | Plasma | 7077 | 562 | 66 | 109 | 301 | 8.5 | 4.6 | 5.2 | 2.8 | 1.9 |
| 3 | F | Het | PDHA1 | 3 | c.225G>T | p.E75D | Plasma | 7461 | 1004 | 101 | 150 | 531 | 9.9 | 5.3 | 6.7 | 3.5 | 1.9 |
| 3 | F | Het | PDHA1 | 3 | c.225G>T | p.E75D | Plasma | 7508 | 676 | 132 | 124 | 482 | 5.1 | 3.7 | 5.5 | 3.9 | 1.4 |
| 4 | M | Hemi | PDHA1 | 4 | c.380G>A | p.R127Q | Plasma | 82 | 863 | 82 | 249 | 343 | 10.5 | 4.2 | 3.5 | 1.4 | 2.5 |
| 5 | M | Hemi | PDHA1 | 5 | c.491A>G | p.N164S | Plasma | 807 | 418 | 286 | 201 | 369 | 1.5 | 1.3 | 2.1 | 1.8 | 1.1 |
| 5 | M | Hemi | PDHA1 | 5 | c.491A>G | p.N164S | Plasma | 476 | 169 | 97 | 73 | 119 | 1.7 | 1.2 | 2.3 | 1.6 | 1.4 |
| 6 | F | Homo | PDHA1 | 6 | c.523G>A | p.A175T | Plasma | 2857 | 561 | 142 | 192 | 194 | 4.0 | 1.4 | 2.9 | 1.0 | 2.9 |
| 7 | F | Het | PDHA1 | 7 | c.616G>A | p.E206K | Plasma | 3 | 796 | 39 | 155 | 615 | 20.4 | 15.8 | 5.1 | 4.0 | 1.3 |
| 8 | M | Hemi | PDHA1 | 7 | c.650C>T | p.P217L | Plasma | 3 | 980 | 20 | 90.5 | 272 | 49.0 | 13.6 | 10.8 | 3.0 | 3.6 |
| 8 | M | Hemi | PDHA1 | 7 | c.650C>T | p.P217L | Plasma | 5 | 1020 | 42.9 | 102 | 603 | 23.8 | 14.1 | 10.0 | 5.9 | 1.7 |
| 8 | M | Hemi | PDHA1 | 7 | c.650C>T | p.P217L | Plasma | 11 | 422 | 127 | 323 | 387 | 3.3 | 3.0 | 1.3 | 1.2 | 1.1 |
| 9 | F | Het | PDHA1 | 7 | c.728A>G | p.Y243C | Plasma | 716 | 1100 | 56 | 141 | 148 | 19.6 | 2.6 | 7.8 | 1.0 | 7.4 |
| 9 | F | Het | PDHA1 | 7 | c.728A>G | p.Y243C | Plasma | 714 | 692 | 102 | 192 | 174 | 6.8 | 1.7 | 3.6 | 0.9 | 4.0 |
| 10 | F | Het | PDHA1 | 9 | c.871G>A | p.G291R | Plasma | 215 | 1130 | 160 | 210 | 210 | 7.1 | 1.3 | 5.4 | 1.0 | 5.4 |
| 11 | F | Het | PDHA1 | 9 | c.874_881dup | p.Met294Ilefs*4 | Plasma | 21 | 1108 | 67 | 136 | 443 | 16.5 | 6.6 | 8.1 | 3.3 | 2.5 |
| 11 | F | Het | PDHA1 | 9 | c.874_881dup | p.Met294Ilefs*4 | Plasma | 30 | 947 | 47 | 178 | 498 | 20.1 | 10.6 | 5.3 | 2.8 | 1.9 |
| 12 | F | Het | PDHA1 | 9 | c.899+1G>C | invariant splice site mut | Plasma | 130 | 1732 | 70 | 185 | 394 | 24.7 | 5.6 | 9.4 | 2.1 | 4.4 |
| 12 | F | Het | PDHA1 | 9 | c.899+1G>C | invariant splice site mut | Plasma | 116 | 1281 | 75 | 191 | 376 | 17.1 | 5.0 | 6.7 | 2.0 | 3.4 |
| 12 | F | Het | PDHA1 | 9 | c.899+1G>C | invariant splice site mut | Plasma | 120 | 1667 | 417 | 2250 | 2500 | 4.0 | 6.0 | 0.7 | 1.1 | 0.7 |
| 13 | F | Het | PDHA1 | 10 | c.904C>T | p.R302C | Plasma | 200 | 1116 | 132 | 204 | 277 | 8.5 | 2.1 | 5.5 | 1.4 | 4.0 |
| 14 | F | Het | PDHA1 | 10 | c.937_940dupAAGA | p.S314KfsX3 | Plasma | 1059 | 237 | 59 | 86 | 105 | 4.0 | 1.8 | 2.8 | 1.2 | 2.3 |
| 15 | F | Het | PDHA1 | 10 | c.985_998dup14 | p.E333DfsX8 | Plasma | 6000 | 419 | 59 | 120 | 126 | 7.1 | 2.1 | 3.5 | 1.1 | 3.3 |
| 15 | F | Het | PDHA1 | 10 | c.985_998dup15 | p.E333DfsX8 | Plasma | 6454 | 441 | 74 | 136 | 138 | 6.0 | 1.9 | 3.2 | 1.0 | 3.2 |
| 16 | M | Hemi | PDHA1 | 11 | c.1132C>T | p.R378C | Plasma | 18 | 2398 | 81 | 174 | 383 | 29.6 | 4.7 | 13.8 | 2.2 | 6.3 |
| 17 | F | Het | PDHA1 | 11 | c.1132C>T | p.R378C | Plasma | 346 | 494.3 | 56.2 | 128.1 | 191.4 | 8.8 | 3.4 | 3.9 | 1.5 | 2.6 |
| 17 | F | Het | PDHA1 | 11 | c.1132C>T | p.R378C | Plasma | 502 | 435.5 | 125 | 201.3 | 248.8 | 3.5 | 2.0 | 2.2 | 1.2 | 1.8 |
| 18 | M | Hemi | PDHA1 | 11 | c.1133G>A | p.R378H | Plasma | 5 | 1448 | 41 | 128 | 450 | 35.3 | 11.0 | 11.3 | 3.5 | 3.2 |
| 19 | M | Hemi | PDHA1 | 11 | c.1133G>A | p.R378H | Plasma | 285 | 2055 | 52 | 146 | 442 | 39.5 | 8.5 | 14.1 | 3.0 | 4.6 |
| 19 | M | Hemi | PDHA1 | 11 | c.1133G>A | p.R378H | Plasma | 284 | 1129 | 36 | 108 | 330 | 31.4 | 9.2 | 10.5 | 3.1 | 3.4 |
| 20 | M | Hemi | PDHA1 | 11 | c.1133G>A | p.R378H | Plasma | 3 | 656.6 | 33 | 71.9 | 191.5 | 19.9 | 5.8 | 9.1 | 2.7 | 3.4 |
| 21 | F | Het | PDHA1 | 11 | c.1142_1145dupATCA | p.W383fs | Plasma | 42 | 1130 | 90 | 200 | 210 | 12.6 | 2.3 | 5.7 | 1.1 | 5.4 |
| 21 | F | Het | PDHA1 | 11 | c.1142_1145dupATCA | p.W383fs | Plasma | 57 | 1030 | 80 | 170 | 320 | 12.9 | 4.0 | 6.1 | 1.9 | 3.2 |
| 21 | F | Het | PDHA1 | 11 | c.1142_1145dupATCA | p.W383fs | Plasma | 67 | 900 | 70 | 140 | 240 | 12.9 | 3.4 | 6.4 | 1.7 | 3.8 |
| 21 | F | Het | PDHA1 | 11 | c.1142_1145dupATCA | p.W383fs | Plasma | 7528 | 580 | 70 | 160 | 160 | 8.3 | 2.3 | 3.6 | 1.0 | 3.6 |
| 21 | F | Het | PDHA1 | 11 | c.1142_1145dupATCA | p.W383fs | Plasma | 11784 | 624 | 91 | 186 | 160 | 6.9 | 1.8 | 3.4 | 0.9 | 3.9 |
| 22 | M | Cpd het | PDHB | 9 & 10 | c.915T>C & c.956A>T | p.C305R & p.D319V | Plasma | 360 | 388 | 111 | 157 | 280 | 3.5 | 2.5 | 2.5 | 1.8 | 1.4 |
| 22 | M | Cpd het | PDHB | 9 & 10 | c.915T>C & c.956A>T | p.C305R & p.D319V | Plasma | 576 | 154 | 107 | 98 | 132 | 1.4 | 1.2 | 1.6 | 1.3 | 1.2 |
| 22 | M | Cpd het | PDHB | 9 & 10 | c.915T>C & c.956A>T | p.C305R & p.D319V | Plasma | 740 | 240 | 546 | 102 | 127 | 0.4 | 0.2 | 2.4 | 1.2 | 1.9 |
| 22 | M | Cpd het | PDHB | 9 & 10 | c.915T>C & c.956A>T | p.C305R & p.D319V | Plasma | 798 | 330 | 52 | 69 | 178 | 6.3 | 3.4 | 4.8 | 2.6 | 1.9 |
| 23 | F | Cpd het | PDHB | 6 | p.426C>T & c.494G>T | p.I142M & p.W165S | Plasma | 391 | 522 | 40 | 108 | 154 | 13.1 | 3.9 | 4.8 | 1.4 | 3.4 |
|  |  |  |  |  |  |  |  |  |  |  |  |  |  |  |  |  |  |
|  |  |  |  |  |  |  |  | **Control max values:** | 449 |  |  |  | 4.3 | 2.8 | 3.49 | 2.91 | 2.1 |
|  |  |  |  |  |  |  | Alanine (Ala), Leucine (Leu), Lysine (Lys), and proline (PRO) values in µM | | | | | | | |  |  |  |
