# Supplementary material for: Utility of specific amino acid ratios in screening for pyruvate dehydrogenase complex deficiencies and other mitochondrial disorders associated with congenital lactic acidosis and newborn screening prospects
Source: JIMD Rep. 2020 Aug 16;56(1):70–81. doi: 10.1002/jmd2.12153 (PMC7653239; doi:10.1002/jmd2.12153)
Supplement: Supplementary file 2 — Supplementary Figure S1 Distribution of plasma Ala, Ala:Leu, and Ala:Lys from PDC deficient subjects due to PDHA1 (20) or PDHB (2) mutations and normal controls. A) Ala (μM), B) Ala:Leu, and C) Ala:Lys from PDC deficient subjects (red dots) and controls (blue dots). Mean and 2 SD (SD) bars shown. Number of false negative (FN) between the maximum value for controls (blue dotted line) and minimum value for PDC deficient subjects (red dotted line) shown. Percentage difference of mean (%Δ), for Ala, Ala:Leu and Ala: Lys metrics were 75%, 122% and 81% respectively. P values were obtained from two‐sample t‐test for un‐pooled variances with one‐tailed significance level of P < 0.05. ***, P < 0.0005. [file JMD2-56-70-s002.pdf]

Suppl. Fig. 1

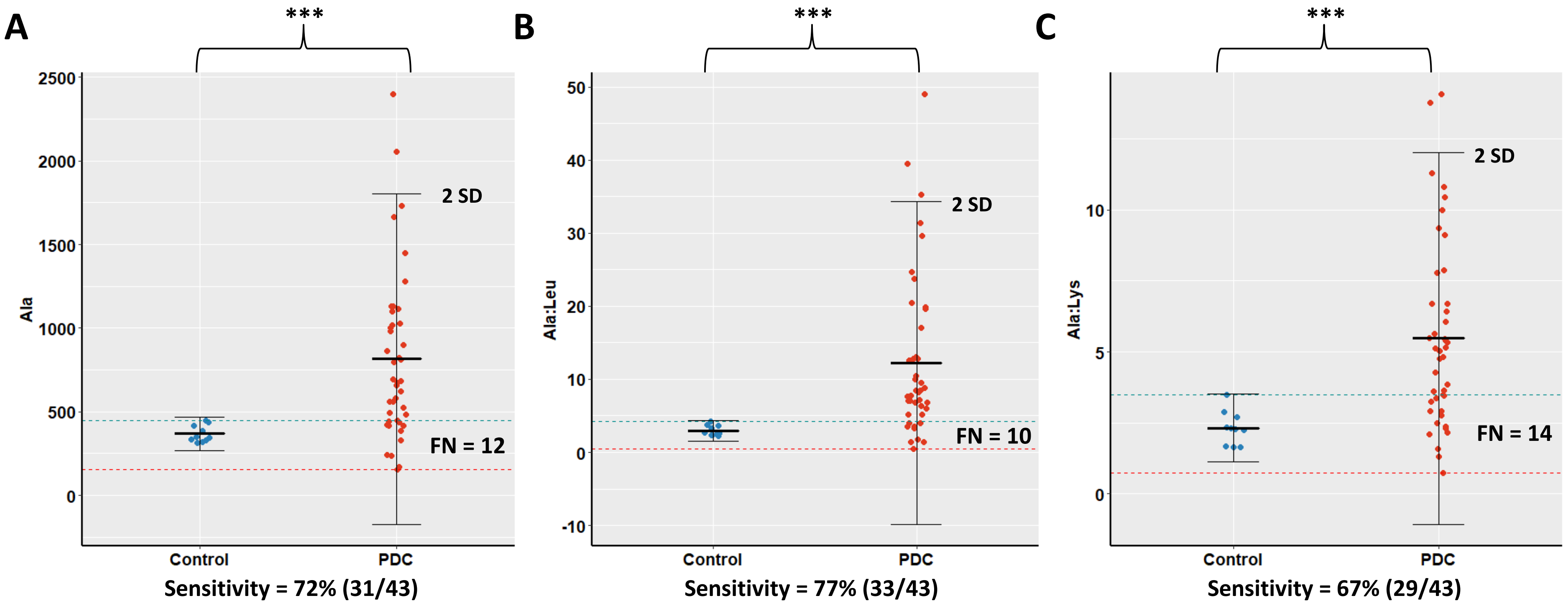

Supplementary Fig. 1. Distribution of plasma Ala, Ala:Leu, and Ala:Lys from PDC deficient subjects due to *PDHA1* (20) or *PDHB* (2) mutations and normal controls. A) Ala (µM), B) Ala:Leu, and C) Ala:Lys from PDC deficient subjects (red dots) and controls (blue dots). Mean and 2 standard deviation (SD) bars shown. Number of false negative (FN) between the maximum value for controls (blue dotted line) and minimum value for PDC deficient subjects (red dotted line) shown. Percentage difference of mean (%Δ), for Ala, Ala:Leu and Ala: Lys metrics were 75%, 122% and 81% respectively. P values were obtained from two-sample t-test for un-pooled variances with one-tailed significance level of $p < 0.05$. ***, $p < 0.0005$.
