# Supplementary material for: Utility of specific amino acid ratios in screening for pyruvate dehydrogenase complex deficiencies and other mitochondrial disorders associated with congenital lactic acidosis and newborn screening prospects
Source: JIMD Rep. 2020 Aug 16;56(1):70–81. doi: 10.1002/jmd2.12153 (PMC7653239; doi:10.1002/jmd2.12153)
Supplement: Supplementary file 4 — Supplementary Table S2 Neonatal and biochemical characteristics of patients fulfilling combined Ala:Leu ≥4.0 and Pro:Leu ≥3.0 criterion (red) from either DBS or plasma analysis at one institution [file JMD2-56-70-s004.pdf]

Supplementary Table 2. Neonatal and biochemical characteristics of patients fulfilling combined Ala:Leu ≥4.0 and Pro:Leu ≥3.0 criterion (red) from either DBS or plasma analysis at one institution

Gender	GA (wk)	BW (g)	DBS Collection time (h)	NBS DBS Ala:Leu	NBS DBS Pro:Leu	Age at PAA Draw #1 (DOL)	PAA Ala:Leu	PAA Pro:Leu	Clinical phenotype and diagnosis	NICU stay at DBS collection	Diet (B/F/BF)
M	38	3060	24	**11.4**	**7.4**	1	**17.1**	**11.5**	HSD10 mitochondrial disease, hemizygous pathogenic *HSD17B10*	Y	B
F	39	3080	25	**7.3**	**4.0**	28	**20.1**	**10.6**	PDCD, heterozygous pathogenic *PDHA1*	Y	BF
M	28	1200	24	**4.4**	**7.1**	61	3.0	2.5	Prematurity, renal disease, transaminitis, bilateral hydrocele, dysmorphic facial features	Y	B
F	41	3210	30	3.4	1.9	2	**5.5**	**3.4**	Jaundice, functional murmur, acidosis	Y	B
F	39	2566	28	3.4	1.9	65	**5.4**	**5.6**	Proximal RTA, biallelic pathogenic *SLC4A4*	N	F
M	37	2330	28	2.2	1.5	6	**5.4**	**3.1**	FTT	N	F
M	29	1050	24	1.3	1.3	16	**4.8**	**3.0**	Prematurity, abnormal NBS methionine, congenital dilation of cerebral ventricles, ASD, twin-twin transfusion syndrome donor twin, multiple congenital anomalies	Y	B
F	27	1210	26	3.1	1.6	35	**4.4**	**3.1**	Prematurity, abnormal NBS methionine	Y	B
M	35	2568	24	2.8	2.4	503	**4.3**	**3.7**	Prematurity, FTT, celiac disease (biopsy proven disease)	N	B or F
F	41	3930	24	2.8	1.8	264	**4.2**	**3.1**	Transcobalamin deficiency, methylmalonic acidemia	N	B
**Average**	35	2420	26	4.2	3.1	98	7.4	5.0			
**Median**	38	2567	25	3.2	1.9	32	5.1	3.3			
**SD**	5	979	2	3.0	2.3	162	6.0	3.3			
**Min**	27	1050	24	1.3	1.3	1	3.0	2.5			
**Max**	41	3930	30	11.4	7.4	503	20.1	11.5			
**n**	10	10	10	10	10	10	10	10			

Abbreviations: ASD, atrial septal defect; BW, birth weight; Diet B, F, or BF refers to breast milk, formula or mix of breast milk and formula, respectively; DBS, dried blood spot; FTT, failure to thrive; GA, gestational age; NBS, newborn screening; PAA, plasma amino acids; RTA, renal tubular acidosis; and SD, standard deviation.
Numbers in red, bold and underlined indicate fulfillment of the combined Ala:Leu ≥4.0 and Pro:Leu ≥3.0 criterion (CRC) and also correspond to the red dots in Fig 2A (for NBS DBS) and Fig 2B (for PAA).
