# Supplementary material for: Utility of specific amino acid ratios in screening for pyruvate dehydrogenase complex deficiencies and other mitochondrial disorders associated with congenital lactic acidosis and newborn screening prospects
Source: JIMD Rep. 2020 Aug 16;56(1):70–81. doi: 10.1002/jmd2.12153 (PMC7653239; doi:10.1002/jmd2.12153)
Supplement: Supplementary file 5 — Supplementary Table S3 Clinical and molecular details of subjects with PDCD and MtDs identified using the protocol [file JMD2-56-70-s005.pdf]

Supplementary Table 3.
Clinical and molecular details of subjects with PDCD and MtDs identified using the protocol

Subject	Genetic change and classification	Molecular testing	Brain anomaly and other clinical features
1	Het *PDHA1* c.1024C>T (p.Arg342*); pathogenic	Targeted NGS panel	Ventriculomegaly, ACC, microcephaly, and seizure
2	*De novo* hemi *HSD17B10* c.85C>G (p.Arg29Gly); likely pathogenic	WES trio	DD. Brain imaging ND
3	Het *PDHA1* c.874_881dup (p.Met294Ilefs*4); pathogenic	15-gene PDCD NGS panel	Cerebellar hypoplasia, ACC, absent cavum septum, pellucidum, hydrocephalus, hypotonia, DD and seizures
4	Compound het *VARS2* c.1925del p.L642Rfs*48 (het father)/c.721C>T p.R241W (het mother); pathogenic/VUS (SIFT/PolyPhen-2, damaging/prob damaging)	WES trio	Normal postnatal brain ultrasound. Brain MRI ND
5	*De novo* het *PDHA1* c.899+2T>A exonic; pathogenic	WES trio	Microcephaly, ventriculomegaly, hearing loss. FTT, and seizure

Abbreviations: ACC, agenesis of corpus callosum; DD, developmental delay; FTT, failure to thrive; het, heterozygous; hemi, hemizygous; MtD, mitochondrial disorder; ND, not done; NGS, next generation sequencing; PDCD, pyruvate dehydrogenase complex deficiency; prob, probably; trio, includes proband and biological parents; WES, whole exome sequencing.
