# Supplementary material for: Utility of specific amino acid ratios in screening for pyruvate dehydrogenase complex deficiencies and other mitochondrial disorders associated with congenital lactic acidosis and newborn screening prospects
Source: JIMD Rep. 2020 Aug 16;56(1):70–81. doi: 10.1002/jmd2.12153 (PMC7653239; doi:10.1002/jmd2.12153)
Supplement: Supplementary file 6 — Supplementary Table S4 Various amino acids and amino acid ratios of subjects with PDCD and MtD identified using the protocol [file JMD2-56-70-s006.pdf]

Supplementary Table 4. Various amino acids and amino acid ratios of subjects with PDCD and MtD identified using the protocol

Blood AA	Table 1 Subjects	DX	Defective Gene	Ala	Pro	Leu	Lys	Glu	Cit	GLY	Ala:Leu	Pro:Leu	Ala:Lys	Pro:Lys	Ala:Glu	Pro:Glu	Ala:Cit	Pro:Cit	Ala:Gly	Pro:Gly
Serum	1	PDCD	*PDHA1*	859	346	33	186	38	14	303	26.0	10.5	4.6	1.9	22.6	9.1	61.4	24.7	2.8	1.1
Plasma	3	PDCD	*PDHA1*	947	498	47	178	152	22	850	20.1	10.6	5.3	2.8	6.2	3.3	43.0	22.6	1.1	0.6
Serum	5	PDCD	*PDHA1*	409	318	31	142	30	9	125	13.2	10.3	2.9	2.2	13.6	10.6	45.4	35.3	3.3	2.5
			Mean	738	387	37	169	73	15	426	19.8	10.4	4.3	2.3	14.2	7.7	49.9	27.6	2.4	1.4
Plasma	2	Other MtD	*HSD17B10*	1354	907	79	358	53	15	839	17.1	11.5	3.8	2.5	25.5	17.1	90.3	60.5	1.6	1.1
Serum	4	Other MtD	*VARS2*	513	252	54	106	48	22	363	9.5	4.7	4.8	2.4	10.7	5.3	23.3	11.5	1.4	0.7
			Mean	934	580	67	232	51	19	601	13.3	8.1	4.3	2.5	18.1	11.2	56.8	36.0	1.5	0.9
NBS DBS	1	PDCD	*PDHA1*	906	415	61	ND	ND	10	436	14.8	6.8	NA	NA	NA	NA	91.4	41.9	2.1	1.0
NBS DBS	3	PDCD	*PDHA1*	370	205	51	ND	ND	12	305	7.3	4.0	NA	NA	NA	NA	31.0	17.1	1.2	0.7
NBS DBS	5	PDCD	*PDHA1*	569	295	83	ND	ND	14	364	6.9	3.6	NA	NA	NA	NA	39.5	20.5	1.6	0.8
			Mean	615	305	65	ND	ND	12	368	9.6	4.8	NA	NA	NA	NA	54.0	26.5	1.6	0.8
NBS DBS	2	Other MtD	*HSD17B10*	1348	879	118	ND	ND	22	694	11.4	7.4	NA	NA	NA	NA	61.7	40.2	1.9	1.3
NBS DBS	4	Other MtD	*VARS2*	357	175	64	ND	ND	16	428	5.6	2.8	NA	NA	NA	NA	22.9	11.2	0.8	0.4
			Mean	853	527	91	ND	ND	19	561	8.5	5.1	NA	NA	NA	NA	42.3	25.7	1.4	0.8

Amino acid concentrations in µM
ND = not done
NA = not applicable
NBS = newborn screening
DBS = dried blood spot
MtD = mitochodnrial disorders
Aa = amino acids
DX = diagnosis
