# Supplementary material for: Utility of specific amino acid ratios in screening for pyruvate dehydrogenase complex deficiencies and other mitochondrial disorders associated with congenital lactic acidosis and newborn screening prospects
Source: JIMD Rep. 2020 Aug 16;56(1):70–81. doi: 10.1002/jmd2.12153 (PMC7653239; doi:10.1002/jmd2.12153)
Supplement: Supplementary file 7 — Supplementary Table S5 Sub‐classification of PDCDs: Currently known, possible and potential molecular etiologies of impaired pyruvate oxidation [file JMD2-56-70-s007.pdf]

Supplementary Table 5.
Sub-classification of PDCDs:
Currently known, possible and potential molecular etiologies of impaired pyruvate oxidation

Enzyme/Complex/Function/Pathway	Gene		
	**Known**	**Possible**	**Potential**
Primary-specific PDCD:			
Pyruvate dehydrogenase complex	*PDHA1, PDHB, DLAT, PDHX*		
Pyruvate dehydrogenase phosphatase	*PDP1*	*PDP2, PDPR*	
Pyruvate dehydrogenase kinase	*PDK3*	*PDK1, PDK2, PDK4*	
Primary-generalized PDCD:			
Multiple 2-ketoacid Dehydrogenases	*DLD*		
Thiamine/thiamine pyrophosphate transporters	*SLC25A19, SLC19A2, SLC19A3*		
Thiamine pyrophosphokinase	*TPK1*		
Pyruvate carrier (mitochondrial)	*MPC1*		
Lipoate metabolism	*LIAS, LIPT1, LIPT2, SIRT4*		
Fe-S Cluster proteins	*BOLA3, NFU1, FDX1L, IBA57, ISCA1, ISCA2, ISCU*	*GLRX5, NUBPL*	*NFS1, LYRM4*
Protease	*LONP1*		
Regulation of glutathionylation			*GLRX2, LRX2*
Secondary PDCD:			
Fatty acid oxidation	*ECHS1*		
Branched-chain amino acid metabolism	*ECHS1, HIBCH*		
Tricarboxylic acid (TCA) cycle	*SUCLA2, SUCLG1*	*SUCLG2, SUCLA1*	
Phosphoenolpyruvate carboxykinase	*PCK2, PCK1*		
Bioenergetics/PDC regulation/TCA cycle	*TAZ*		
Other:			
Pyruvate carboxylase	*PC*		
	GOT2, KMT2B, SLC25A1	*GOT2, FBXL4*	
